# Report on the Medical Uses of Carbolic Acid

**Published:** 1871-07

**Authors:** N. S. Davis

**Affiliations:** Chicago, Ill., Chairman of the Committee


### REPORT ON THE MEDICAL USES OF CARBOLIC ACID.

By N. S. DAVIS, M.D., Chicago, Ill., Chairman of the Committee.

Read to the Illinois State Medical Society, at the Annual Meeting in Peoria, May 16, 1871.

At the last Annual Meeting of this Society, the undersigned were appointed a Special Committee to report on the medical uses or value of the carbolic acid in the treatment of diseases when taken internally. It would doubtless have been more satisfactory to the Society if we could have collated, in the form of a report, the results of the clinical experience of many members of the profession, gathered by a close examination of our medical literature; but our time has been so closely occupied that we have been unable to do this. We shall, therefore, attempt nothing more than a plain statement of our own experience in regard to the use of the remedy.

It is about three years since carbolic acid began to attract attention as a remedy for internal administration. The kindred substance, creosote, had long be known, and used to a limited extent in the treatment of some forms of disease. But the carbolic acid, in its pure crystalline form, was first introduced to the notice of the profession as an external application in surgical injuries, for the purpose of destroying living germs, and preventing suppuration or putrefaction. It was not until it had been extensively used for these purposes in surgical practice, and the idea had become quite prevalent that various diseases, classed as zymotic, depended on the presence and action of living germs, either animal or vegetable, that it was suggested as a remedy of probable efficacy in the treatment of such diseases. During the brief period which has elapsed since this suggestion was made, the carbolic acid has been used as an internal remedy, more or less, in the treatment of all forms of intestinal flux, from the simple infantile diarrhœa to the epidemic cholera and dysentery; in the low or typhoidal types of fever; in all the forms of angina, from simple catarrhal inflammation to scarlatina anginosa and diphtheria; and in some of the varieties of carcinoma.

During the last two years, we have prescribed the carbolic acid very often, and in a considerable number of morbid conditions. In the various grades of irritation or morbid sensitiveness of the mucous membranes of the alimentary canal, especially in children, we have found it a very valuable remedy. A few cases will serve to illustrate more fully the application of the remedy than we could convey in any other manner.

Case 1st.—A. B., child eight months old, nursing. The bowels had been slightly loose for three or four days, the discharges thinner and more offensive than natural, but not more than three times a day, until July 3d, 1870, when it began to have active diarrhœa, the discharges being very thin and of a greenish color, accompanied by a prompt rejection of whatever it took into its stomach, either by nursing or drinking. It was not the active vomiting of severe cholera morbus, but that morbid sensitiveness of the stomach that causes rejection of the ingesta and serious diarrhœa. There was no febrile reaction, but rather paleness and coolness of the surface. The mother was directed to let the child nurse often, but only a little at a time, and to give it no drinks except one or two teaspoonfuls at a time of cold water or mucilage; and the following prescription was given:

℞. Carbolic Acid Crystals ---------------3grs.
Glycerine---------------------------℥ss.
Camph. Tinct. Opii. -----------------℥ss.
Water ------------------------------℥ij.

Mix, and give 20 drops every two hours until the stomach and bowels are quiet. When there have been no evacuations up or down for twelve hours, then extend the interval between the doses to three hours. Under this treatment the vomiting ceased during the first twelve hours, but moderate diarrhœa continued, and the medicine was also continued at intervals of three hours. On the third day after commencing the treatment there was no vomiting, and only two intestinal evacuations more healthy in character. The same medicine was continued four times a day for three days longer, when the child appeared well, and treatment was discontinued. During the summer of 1870, we treated more than 70 cases similar to the one just related, embracing children from six months to two years of age, with the same formulæ, and nine out of every ten speedily recovered. Such of the children as had been weaned were fed on small but frequently repeated doses of a thin porridge, made of sweet milk and wheat flour. In a very few instances the medicine appeared to exert no influence over either the vomiting or diarrhœa, and other remedies were made available. It will be remembered that the cases here alluded to were recent and simple in their nature. The following will illustrate another class of cases, of greater severity, and of very frequent occurrence during the months of July, August, and September.

CASE 2d.—*July 27th.* Called to C. T——'s child, aged 15 months, still nursing. The child had commenced to have moderate diarrhœa, or "summer complaint," as it is termed, during the first week in July, which had continued, with only occasional vomiting when it took too much into its stomach, until the 24th. It had become pale and thin in flesh, but still most of the time cheerful, and the mother,. as is usual in such cases, attributing the looseness to "teething," had used no remedies except one or two doses of castor-oil. During the night of the 24th, the child became more restless, the bowels moving every two or three hours, and the stomach promptly rejecting whatever was taken into it. The intestinal discharges were very thin, yellow, and offensive. The following day a physician was called, who prescribed suitable doses of anodyne and alterative powders, mustard sinapisms over the epigastrium, and the next day some laxative mixture, sufficient to move the bowels. Almost every dose of the medicine, however, was rejected by vomiting, and the original symptoms continued without abatement. When we were called on the 27th, the child was much emaciated, the countenance haggard, extremities cool, pulse quick and feeble, paroxysms of great restlessness, with intervening somnolency—almost every paroxysm of restlessness ending in a discharge from the bowels of a greenish yellow color, and almost as thin as water, with little specks of mucus in it. There was pretty uniform vomiting within a few moments after nursing or taking any kind of drink. The urinary secretion was very scanty. We advised the mother to let the child nurse only a little at a time, but often, and to give no other drink except teaspoonful doses of ice-cold water, of which it was very fond. For medicine, we directed the following:

℞. Carbolic Acid Crystals ............... 3grs.
Glycerine (pure) ...................... ℥ss.
Water ................................. ℥ijss.

Mix, and give half a teaspoonful every hour until the vomiting ceases, and the breast milk is retained well. Also the following:

℞. Nitrous Ether ......................... ʒss.

Camph. Tinct. Opii. .................. ʒss.

Mix. Give 20 drops in half a tablespoonful of sweetened water every three hours, to help allay the irritability of the bowels, and promote more active secretion of the kidneys.

*July 28th.*—The vomiting has nearly ceased; the evacuations from the bowels are less frequent, but nearly the same in character, and the urine only slightly increased in quantity. Ordered both prescriptions continued, but the solution of carbolic acid only every three hours, making it come alternately with the paregoric and spirits of nitre.

*July 29th.*—Child nurses well, and retains all it takes into its stomach; countenance much improved; urine more abundant; but the intestinal discharges continue to occur every three or four hours, and remain thin and pretty copious. Directed the carbolic acid solution to be continued every six hours, and half-way between, one of the following powders, *viz.:*

℞. Sub. Nit. Bismuth..................12grs.
Pulv. Geranium Root ............... 4grs.
Pulv. Doveri........................ 1gr.

Mix. Divide into six powders.

Under this treatment the bowels steadily improved, and on the 1st of August the carbolic acid was omitted, and only one powder given each night and morning; and after three days more they were dispensed with altogether, the child needing no further treatment. As already remarked, this case is the representative of a large number that were treated, and in nearly all of which the carbolic acid was of great service in allaying the gastric irritation and vomiting, but in all, or nearly all of which, other remedies were required to aid in restoring a healthy condition to the bowels. In the first stage of active cholera morbus, both in children and adults, we have many times promptly arrested the active symptoms by using the following formulæ:

℞. Carbolic Acid Crystals ...............6grs.

Glycerine ............................℥ss.

Camph. Tinct. Opii...................℥jss.

Water.................................℥ij.

Mix. Give to adults one teaspoonful every half hour or hour until the symptoms are relieved, and doses proportionately less for children. In active dysentery or acute inflammation of any part of the mucous membrane of the alimentary canal, we have found little or no advantage from the use of carbolic acid, but in many cases of chronic dysentery, accompanied by flatulency and gastric irritability, it has afforded much relief when given with paregoric, as in the last formulæ stated above, and repeated every three, four, or six hours.

Fevers.—The theories that, on the one hand, attribute typhoid fever to the action of some specific animal poison capable of producing general febrile disturbance, in connection with a specific morbid action in the glands of the mucous membrane of the intestines, and, on the other, make the influence of carbolic acid depend on its power to destroy such poisons, very naturally suggest the latter as a remedy for the former. Our own clinical experience, however, does not lead us to place much confidence in the efficacy of carbolic acid as a leading remedy in the treatment of this or any other variety of general fever. And yet there are conditions that sometimes arise during the progress of both typhoid and typhus fevers that may be met more effectually by carbolic acid than by any other remedy that we have tried. It has occasionally happened, though not often, that these fevers have been accompanied, in the early stage, by such a morbid sensitiveness of the stomach, that almost everything taken into it was rejected by vomiting, thereby interfering seriously with the proper administration of both food and medicine. In several such cases we have given small and frequently repeated doses of the solution of carbolic acid, with the most happy effect in relieving the gastric irritation and modifying the the course of the fever. It also sometimes happens, in the advanced stage of these fevers, even after many of the symptoms of convalesence have become apparent, that a very troublesome sensitiveness of the stomach and bowels spring up, interfering with our efforts to nourish and sustain the patient at a very critical period of his progress. The following case will illustrate the point we have in view better than any description we could give:

Case 3d.—*Feb. 10th, 1871.* Miss G——, a German servant girl, aged about 20 years, had been admitted into the Mercy Hospital ten days previously, then in the beginning of the third week of a severe enteric typhus fever. At the time of her admission her expression of countenance was dull; face suffused with a dark flush; lips, mouth, and tongue dry, and the latter red at the edges, with a brown, dry coat over the middle; skin generally moderately hot and dry; pulse 120 per minute, and soft; dry bronchial ronchi over the whole chest, with oppressed breathing; abdomen full and tympanitic, with from four to six thin, brown, and offensive intestinal discharges every twenty-four hours; mind dull, wandering, and indifferent. Could not learn whether she had been subject to any medical treatment or not. She was placed on the use of an emulsion of oil of turpentine and tincture of opii every four hours, with six grains of chlorate of potass, and six drops of hydrochloric acid in mucilage of gum arabic between. For nourishment, she was to take two tablespoonfuls of sweet milk and wheat flour porridge every two hours, with the same quantity of beef tea or essence between. After the first four days the more important symptoms began to abate. The surface was less hot and dry; the mind less wandering; the abdomen less tympanitic; the mouth and tongue less dry; the dry bronchial ronchi were displaced by slight moist ronchi, and a little expectoration; the pulse was only 100 per minute; and the intestinal discharges not more than two or three in the twenty-four hours. These improvements continued through the fifth and sixth, and were so marked as to create the impression that convalesence had already commenced, and the medicines were given at longer intervals, and the quantity of nourishment moderately increased. On the seventh, however, the stomach began to reject both food and medicine, and the intestinal evacuations became more frequent, with increasing tympanities. Both the emulsion and the chlorate solution were discontinued, and instead a powder of sulphate of morphia 1-6gr., and sub. nit. bismuth 6grs. was directed to be given every two hours until the stomach and bowels were quieted. On the following day, finding that the powders had been rejected by vomiting, and no improvement in the other symptoms, they were continued at longer intervals, and a teaspoonful of the following mixture given every two hours:

℞. Aromat. Spts. Ammonia ---------------ʒiij.

Camph. Tinct. Opii -------------------℥jss.

Simple Syrup -------------------------℥ss.

Aqua Camph. --------------------------℥j.

Mix. Sinapisms were applied over the epigastrium. On the 10th, the patient was found not only no better, but decidedly worse. The countenance was haggard, the skin cool, the pulse frequent and feeble, the abdomen much distended and tense, intestinal discharges frequent, and very thin; stomach still rejecting both food and medicine, and yet the tongue was clean and the mouth free from sordes, and the mind clear. All previous medicines were omitted, and the patient rigidly restricted to a single tablespoonful of thin milk porridge every half hour, and one teaspoonful of the following prescription every hour:

℞. Carbolic Acid Crystals ---------------6grs.

Camph. Tinct. Opii -------------------℥iss.

Glycerine ----------------------------℥ss.

Aqua ---------------------------------℥ij. M.

Before the end of twenty-four hours the vomiting had ceased, and the intestinal discharges were less frequent. The medicine was continued every two hours, with nourishment as before. From this time the patient continued steadily to improve, and the interval between the doses of medicine was lengthened from day to day, and the nourishment given in larger quantities, and at larger intervals, and on the 18th of the same month she was fully convalescent.

Among the poorer classes of society, cases of this variety of fever are often neglected in the early stage, and several have come under observation in which an extreme irritability of the stomach has been developed in the advanced stage, and in which the carbolic acid, given as above, afforded speedy relief.

We have given the carbolic acid in solution in water, with a little glycerine, as in the following formulæ, in many cases of indigestion, characterised by fœtid eructations, occasional pains in the abdomen, especially soon after eating, and one or two offensive discharges from the bowels each day.

℞. Carbolic Acid Crystals--------------6grs.
Glycerine (pure)---------------------℥ss.
Water-------------------------------℥iijss. M.

Take one teaspoonful just before each meal, and at bedtime, and in nearly all the cases with decided benefit. We have also used the same formulæ, in doses suited to the age of the patient, in several cases of scarlatina anginosa, diphtheria, and catarrhal sore throat, and generally with benefit, especially in the ulcerative stage of those affections. We have used the same solution in six cases of unusually severe vomiting in the early months of pregnancy. Two of these cases were unusually severe, and had persisted until past the fifth month, when they came under my observation. Both were first pregnancies, and the patients had become extremely reduced in flesh and strength, from inability to retain nourishment. They had tried sub. nitrate bismuth, oxalate of cerium, lupulin, and a variety of antacids, and some stimulants, but without relief. Both, however, were speedily relieved by taking one teaspoonful of the solution of carbolic acid as above given; at first, every three hours, and after the vomiting was checked, the remedy was continued before each meal for several weeks. The remaining four cases were less severe. In two of them the medicine taken before each meal afforded considerable relief, but did not entirely stop the nausea; while in the remaining two it produced not only no beneficial effect, but seemed to be especially repugnant to the patients.

Cancer.—Two cases of cancerous disease and ulceration of the os and neck of the uterus were much relieved by using the solution of carbolic acid internally, at each meal time, and a stronger solution twice a day as vaginal wash. They were kept comparatively comfortable for many months, but the effects were only palliative.

A neighboring physician told us he had a case of cancerous disease, of well-marked character, that had been kept stationary and the patient comfortable more than twelve months, under the constant use of carbolic acid. We have also used it with temporary benefit in two cases of cancerous disease of the stomach.

From the foregoing details, it will be seen that we do not regard carbolic acid as a specific for the cure of any form of disease, but from its mildly sedative influence on the organic nervous system and mucous surfaces, coupled with strong antiseptic properties, it is admirably adapted to meet certain indications that arise during the progress of a great variety of diseases. We might easily extend this report by further details, including the local application of carbolic acid to various cutaneous eruptions, but enough has been given to indicate clearly the directions in which it may be applied and administered with reasonable expectation of benefit.

N. S. DAVIS,

